# Apparent Half-Lives of Hepta- to Decabrominated Diphenyl Ethers in Human Serum as Determined in Occupationally Exposed Workers

**DOI:** 10.1289/ehp.8350

**Published:** 2005-09-21

**Authors:** Kaj Thuresson, Peter Höglund, Lars Hagmar, Andreas Sjödin, Åke Bergman, Kristina Jakobsson

**Affiliations:** 1 Department of Environmental Chemistry, Stockholm University, Stockholm, Sweden; 2 Competence Center for Clinical Research and; 3 Department of Occupational and Environmental Medicine, Lund University Hospital, Lund, Sweden

**Keywords:** BDE-209, BFR, brominated flame retardants, deca-BDE, half-life, human exposure, PBDEs, polybromodiphenyl ethers

## Abstract

The aim of the present study was to model apparent serum half-lives of polybrominated diphenyl ethers (PBDEs) with 7–10 bromine substituents. Workers with occupational exposure to PBDEs have elevated serum levels of PBDEs, but these substances are also found in the general population and are ubiquitous environmental contaminants. The calculations were based on exposure assessments of rubber workers (manufactured flame-retarded rubber compound) and electronics dismantlers who donated blood during a period with no work-related exposures to PBDEs, and referents without any known occupational exposure (clerks, cleaners, and abattoir workers). The workers had previously been found to have elevated levels of high- and medium-brominated diphenyl ethers compared with the referent populations. We performed nonlinear mixed-effects modeling of kinetics, using data from previous and present chemical analyses. The calculated apparent half-life for decabromodiphenyl ether (BDE-209) was 15 days (95% confidence interval, 11–18 days). The three nona-BDEs and four octa-BDE congeners were found to have half-lives of 18–39 and 37–91 days, respectively. BDE-209 has a short half-life in human blood. Because BDE-209 is commonly present in humans in general, the results of this study imply that humans must be more or less continuously exposed to BDE-209 to sustain the serum concentrations observed. BDE-209 is more readily transformed and/or eliminated than are lower brominated diphenyl ether congeners, and human health risk must be assessed accordingly.

Polybrominated diphenyl ethers (PBDEs) have for decades been applied as additive flame retardants in polymers in electronics and electric goods, and in rubber, plastics, and textiles (Alaee et al. 2003). The technical mixture of decabromodiphenyl ether, in which BDE-209 is the major congener, now dominates the market among the PBDE products [Bromine Science and Environmental Forum (BSEF) 2004]. The concentrations of PBDEs, including BDE-209, have accumulated in humans and the environment worldwide (Gill et al. 2004; Hites 2004; Law et al. 2003). Hitherto, mainly PBDE congeners with four to six bromines have been assessed in human blood and milk (Fängström et al. 2005; Hites 2004; Sjödin et al. 2003). A few studies also included exposure to higher brominated PBDE congeners, both in humans occupationally exposed to PBDEs (Jakobsson et al. 2002; Sjödin et al. 1999; Thuresson et al. 2005) and in humans exposed to background concentrations (Fängström et al. 2005; Schecter et al. 2003; Thuresson et al. 2005).

It is not yet fully understood how humans are exposed to the PBDEs, but ingestion (food and dust) and inhalation seem to be important routes of exposure. Many PBDEs have been reported to be present in food (Bocio et al. 2003; Domingo 2004; Huwe and Larsen 2005; Tittlemier et al. 2004), in ambient and occupational air (Butt et al. 2004; Pettersson-Julander et al. 2004; Sjödin et al. 2001), and in household dust (Schecter et al. 2005; Stapleton et al. 2005; Wilford et al. 2005). Hence, dominant PBDE exposure routes differ from those of major historical persistent organic pollutants [e.g., polychlorinated biphenyls (PCBs) and 2,2-bis(4-chlorophenyl)-1,1,1-trichloroethane (DDT)] because there are numerous indoor sources for PBDEs but many fewer for the PCBs and DDT.

All PBDEs are bioavailable, but the chemical and biologic properties of the congeners vary, as reviewed recently by Birnbaum and Staskal (2004), Darnerud (2003), and Gill et al. (2004). Based on knowledge of the kinetics of halogenated aromatics, we should expect great variations in their degradation and excretion rates. Geyer et al. (2004) recently presented estimated half-lives based on experimental data for a number of brominated flame retardants, including tetra- to hexa-BDEs, but not for any higher BDEs. These low- to medium-brominated PBDE congeners were indicated to have very slow elimination rates. A similarly long half-life for 2,2′,4,4′,5,5′-hexabromobiphenyl has also been reported (Lambert et al. 1990). In contrast, observations in five electronics dismantlers sampled before and after 30 days of vacation suggested a rather rapid elimination of BDE-209, with a median decrease of 66% during this period of time (Sjödin et al. 1999). This was the first observation that BDE-209 is degraded or eliminated at a much higher rate than lower brominated congeners of PBDEs. Similarly, Sandholm et al. (2003) observed a short half-life (*t*_1/2_ = 2.4 days) for BDE-209 in rats dosed with this congener. Also, gray seals experimentally dosed with BDE-209 indicated a short half-life (8–13 days) of this compound (Thomas et al. 2005).

The objective of the present study was to calculate the apparent half-lives for BDE-209 and other higher BDEs in human serum, using data from occupationally exposed workers sampled before, during, and after a vacation period. If we assume that those who are known to be occupationally exposed to PBDE do not experience any other PBDE exposure beyond normal background outside their work environment, we can determine apparent half-lives of compounds with reasonably short half-lives compared with the duration of the vacation. Because two types of exposure have to be considered, work-related and general background exposure, we also used data from reference populations without any known occupational exposure for estimation of background exposure levels.

## Materials and Methods

### Study groups and sampling.

We included five groups of workers in our study; these groups have all been previously described in detail (Sjödin et al. 1999; Thuresson et al. 2005). The serum concentrations of some PBDEs in these groups (cross-sectional investigations) are summarized in Table 1. For this study, we analyzed an additional number of samples from workers who donated blood samples during their vacations from the electronics dismantling plant (Sjödin et al. 1999) and from the rubber manufacturer (Thuresson et al. 2005).

#### Electronics dismantlers.

Workers in a recycling plant performing manual dismantling of electronic goods were shown by Sjödin et al. (1999) to have elevated serum concentrations of BDE-47, BDE-153, BDE-183, and BDE-209 compared with referents. In the present study, three female and one male dismantler, 43–49 years of age, were sampled immediately before the summer vacation and at the end of their vacation, 28 or 29 days later. In addition, three or four samples were obtained from each worker during their vacation period. The sampling took place in 1998, 1 year after the first study (Sjödin et al. 1999).

#### Workers manufacturing flame-retarded rubber compound.

At a plant manufacturing technical rubber, batches of deca-BDE flame-retarded rubber compound were regularly produced, usually during campaigns of 1–2 days, two to four times a month. The commercial deca-BDE used as an additive consisted of mainly BDE-209 with traces of nona-BDEs and octa-BDEs. The mixers had elevated serum levels of octa-BDEs to BDE-209, whereas the millers had serum levels comparable with male referents (Thuresson et al. 2005). In the present study, we include blood samples from four male rubber mixers, 25–60 years of age, and three male millers, 31–45 years of age, drawn immediately after a period of production of flame-retarded rubber in June 2000, on the day before the start of the summer vacation, and again at the end of vacation (31, 34, or 43 days later). Additionally, we obtained between one and seven samples from each of the workers during their vacation.

In 2002, the production of flame-retarded rubber had decreased substantially at the plant, and no batches were produced between the end of September and the end of November that year. Two mixers were resampled on the morning before a 1-day production period and at days 2 and 7 after the handling of deca-BDE. Data from these samplings were not included in the modeling of half-lives but are reported separately.

#### Clerks.

In June 1997 blood samples were obtained from 20 female clerks, 25–61 years of age, regularly using computers 8 hr/day (Sjödin et al. 1999). Samples from their vacation period were not obtained.

#### Cleaners.

In September 1997, blood samples were obtained from 20 female hospital cleaners 30–60 years of age (Sjödin et al. 1999). Their work was performed without any computer support, and their electrical and flame-retarded environment was considered to be at a minimum. They all had none or very limited computer experience. Samples from their vacation periods were not obtained.

#### Abattoir workers.

Blood samples were obtained from 18 male abattoir workers 24–60 years of age in March 2000 (Thuresson et al. 2005). Their work was performed without any computer support, and the electrical and flame-retarded environment was considered to be at a minimum at the plant. They all had none or very limited computer experience. No samples from any vacation periods were obtained.

#### Blood sampling.

Blood was drawn from the cubital vein into evacuated plain tubes (Vacutainer; Becton Dickinson Vacutainer Systems, Rutherford, NJ, USA). The serum was centrifuged, transferred to dark-colored acetone-washed glass bottles, frozen, and kept at –20°C until the start of the chemical analysis. Informed consent was obtained from all subjects, and the study was approved by the Ethics Committee at Lund University, Sweden (protocol LU 227-97).

### Analysis of PBDEs.

#### Chemicals and instruments.

Solvents, reference standards, and other chemicals used in analysis of serum samples, as well as the instrumental support of the work, have been reported previously (Sjödin et al. 1999; Thuresson et al. 2005).

#### Analyses of human serum.

**Figure 1:**
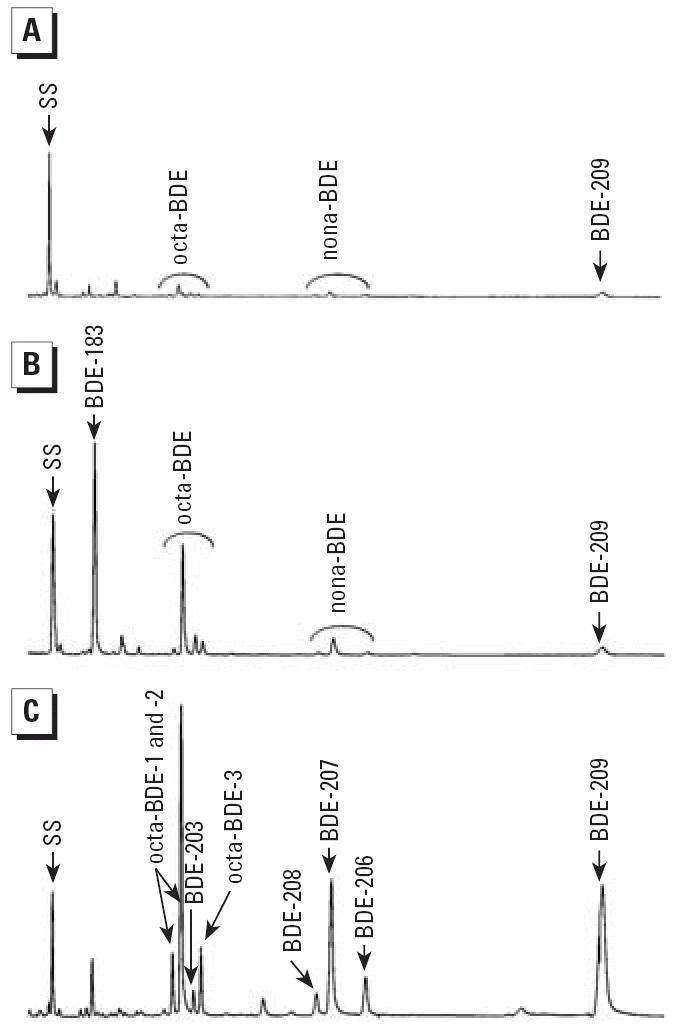
Representative ECNI mass chromatograms with SIM of the *m*/*z* = 79 and 81 ions presented for a referent (*A*; an abattoir worker without known occupational PBDE exposure), an electronics dismantler (*B*), and a rubber mixer (*C*). BDE-183 is the dominating peak in the dismantler (*B*), and octa-BDE congeners to BDE-209 dominate in the rubber mixer (*C*). The peak heights of the surrogate standard (SS; BDE-138) are approximately the same in *A*–*C*.

Extraction of serum, lipid determination, and partitioning between an organic solvent and aqueous sodium hydroxide have been described in detail elsewhere (Hovander et al. 2000; Sjödin et al. 1999; Thuresson et al. 2005). We performed the cleanup procedure with sulfuric acid lipid removal, and quantitative analyses of PBDEs by gas chromatography–mass spectrometry [electron capture negative ion (ECNI) monitoring, *m*/*z* = 79, 81] as previously described in detail for the individual studies (Sjödin et al. 1999; Thuresson et al. 2005). We used the response factor for BDE-203 for quantification of structurally unidentified octa- and nona-BDEs, which probably underestimate the nona-BDE levels. For all other PBDE congeners, authentic reference standards were available. Selected ion monitoring (SIM) chromatograms of the PBDE peak patterns in a referent (abattoir worker), an electronics dismantler, and a rubber worker are presented in Figure 1 and show the differences in their PBDE patterns.

The blood samples were analyzed at different time points between 1998 and 2004. All samples from each study group were analyzed together. Because the original limit of quantification (LOQ) varied between data sets, we had to recalculate uniform LOQs for the present study. For all serum samples analyzed, we used a signal-to-noise (S:N) ratio > 5 to define the LOQ, when no interference was present in the blank samples. If interferences were present in the blank samples, the amount of the analyte in the sample had to be at least five times the average blank level to be accepted. The amounts of PBDEs in blank samples, if any, were subtracted from sample concentrations before being reported. When LOQs differed between sets of data recorded and analyzed at different time points, the LOQ was set as the highest measured LOQ (based on S:N ratio or blank sample levels) or the lowest quantified value in all data sets.

### Determination of apparent half-lives.

For modeling of the half-life of each PBDE congener, we assumed that each subject had a certain, constant non-work-related exposure independent of profession. Each of the occupationally exposed workers also had a work-related exposure considered to be at a steady state before vacation but different between groups and subjects. Considering the start of the vacation as day 0, Equation 1 for each subject can be expressed as follows:


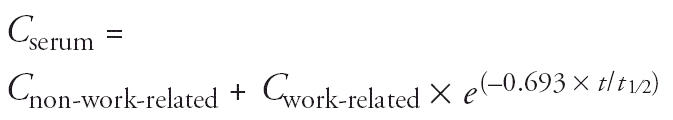


Based on the results from the cross-sectional studies, we selected groups for inclusion in the models. Clerks, cleaners, and abattoir workers were considered occupationally unexposed in all half-life calculations. We considered the electronics dismantlers to be occupationally exposed to all PBDE congeners studied. Four rubber mixers were considered to be occupationally exposed to deca-, nona-, and octa-BDEs but not to lower brominated congeners. The remaining three rubber millers had PBDE concentrations that did not differ from the referents and were considered occupationally unexposed.

The rubber millers and abattoir workers (all male) were sampled in 2000, whereas the female referents were sampled in 1997. Thus, we added a separate addition factor for millers and abattoir workers to the model (whether it represents a calendar year effect or a sex difference cannot be distinguished). For BDE-209 the inclusion of this addition factor yielded a better fit of the model but did not change the half-life estimate. For all other congeners, there were no differences in the model fits, and this additional factor was set to zero.

In total, 107 observations from 68 subjects for BDE-209 and BDE-183 were available for the calculations. Because octa- and nona-BDEs had not been determined for cleaners and clerks, 67 observations from 28 individuals were available. For serum concentrations below the uniform LOQ, the level was set to LOQ/2, and the values could thus be used in the kinetic calculations.

We used NONMEM (version 5, level 1.1; Beal and Sheiner 1982) for the calculations. We determined the variance in the populations, expressed as a coefficient of variation for both the non-work-related and work-related exposures. Approximate estimations of the 95% confidence interval (CI) were calculated as the estimate ± 2SE of the estimate.

Additionally, we performed a nonlinear regression analysis of concentration versus time data between days 10 (to ensure that the uptake phase had ended) and 34 in two rubber workers with a rich data set. We assumed a monoexponential decline on top of the baseline (background) level. We used NLIN Procedure in SAS (version 8.2; SAS Institute, Cary, NC, USA).

## Results

PBDE serum concentrations from four electronics dismantlers and four rubber mixers sampled two to seven times from the beginning to the end of their vacation are given in Table 2. The half-lives of the higher BDEs increased with decreasing number of bromine substituents (Table 3). For BDE-209, the calculated *t*_1/2_ was 15 days (95% CI, 11–18 days). For the three nona-BDEs (BDE-206, BDE-207, and BDE-208), the calculated *t*_1/2_ values were 18, 39, and 28 days, respectively, with the shortest half-life for the PBDE con-gener with three *ortho*-bromines (BDE-206). The four octa-BDE isomers (octa-1, octa-2, BDE-203, and octa-3) had calculated half-lives of 72, 85, 37, and 91 days, respectively, with the shortest half-life for BDE-203. BDE-183 had a calculated half-life of 94 days (95% CI, 68–120 days). The apparent half-lives for BDE-153 and several other medium- and low-brominated congeners could not be determined but were indicated to be much longer than our 1-month observation period (BDE-153 data in Table 2).

**Figure 2:**
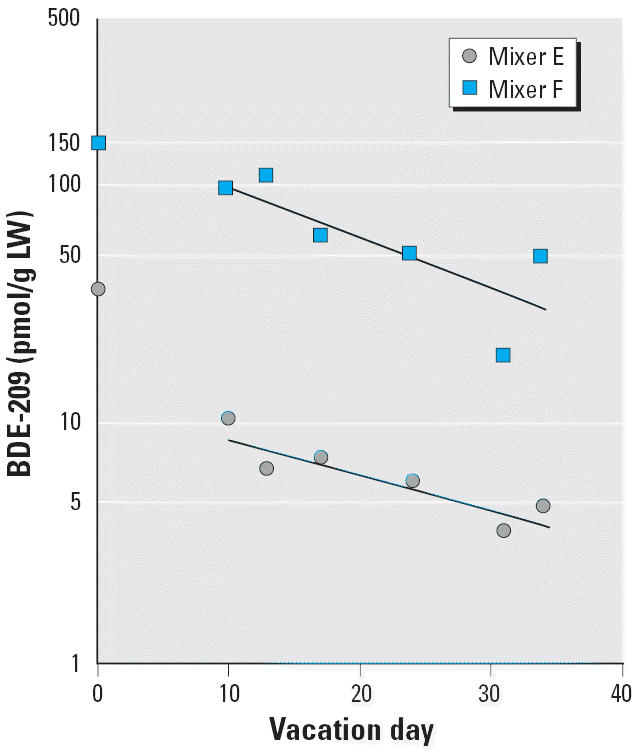
Observed serum concentrations [pmol/g lipid weight (LW)] of BDE-209 during vacations in two rubber mixers. The lines indicate the predicted decline between days 10 and 34. The corresponding calculated half-lives were 14 days for mixer E and 16 days for mixer F.

In two rubber mixers with a rich data set, a traditional elimination model yielded similar results for BDE-209, with half-lives of 14 and 16 days, respectively, calculated between days 10 and 34, to ensure that the uptake phase was over (Figure 2).

**Figure 3:**
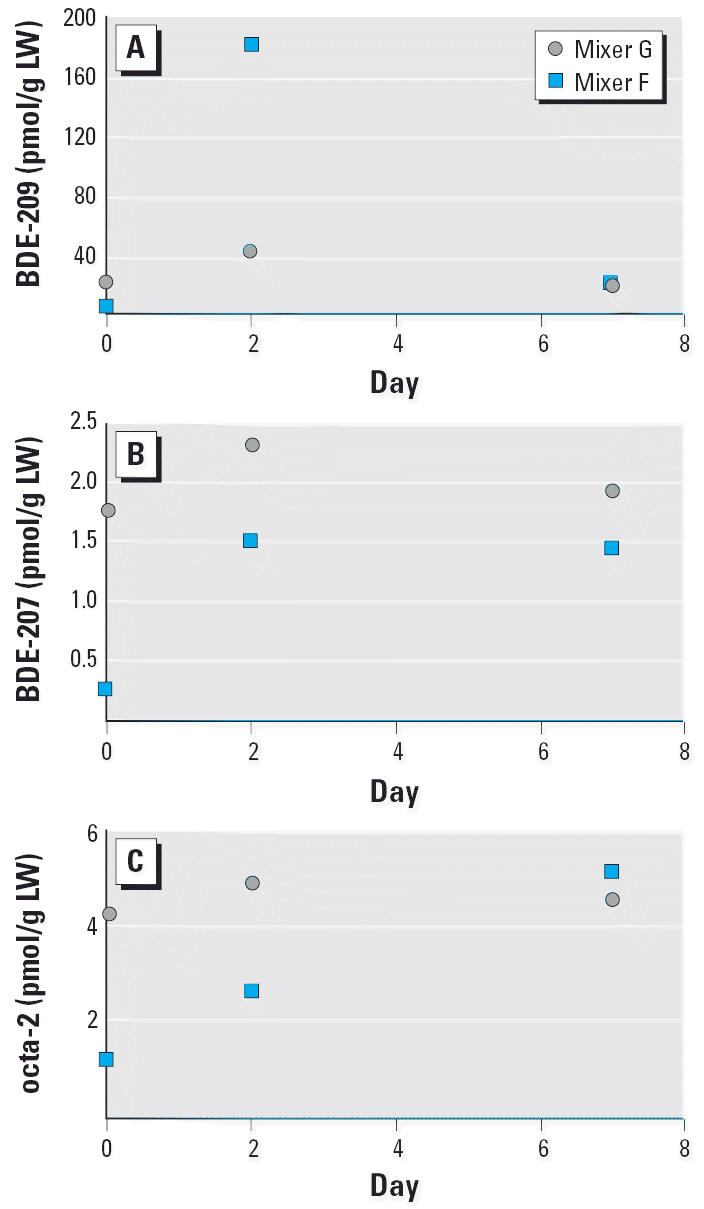
Serum concentrations [pmol/g lipid weight (LW)] of BDE-209 (*A*), BDE-207 (*B*), and one not yet identified octa-BDE congener (*C*) in two rubber mixers immediately before work-related exposure to deca-BDE during 1 work day (day 0), and 2 and 7 days after the end of exposure.

We observed a rapid uptake of BDE-209 after an isolated 1-day period of deca-BDE exposure during production of a batch of flame-retarded rubber (Figure 3). The levels of BDE-209 increased between days 0 and 2 and had clearly declined at day 7 in both workers. In contrast, the octa-BDE levels were at their highest at day 7. For nona-BDE, only a slight decrease between days 2 and 7 was observed. Moreover, the relative increase of BDE-209 between day 0 and day 2 was higher than the relative increase of the octa-and nona-BDEs in both workers.

## Discussion

Initial findings in a small number of electronics dismantlers had indicated that the half-life of BDE-209 might be shorter than the half-lives of PBDEs with a lower degree of bromination (Sjödin et al. 1999). In the present study, we used a larger number of individual measurements from studies of occupationally exposed subjects and referents without known occupational exposure to PBDEs to further investigate PBDE congener half-lives (Sjödin et al. 1999; Thuresson et al. 2005). We found that the apparent half-lives of deca- to hepta-BDEs in serum increased with decreasing number of bromine substituents. For the fully brominated BDE-209, the apparent half-life was as short as 15 days. Data from two different types of modeling agreed well. The results obtained from the present study thus confirm our first observation in electronics dismantlers (Sjödin et al. 1999) and are corroborated by observations in two rubber workers after a single day of exposure to deca-BDE 16 months later (Figure 2). Thus, it is not likely that fluctuations in background exposure levels can invalidate our findings. Our data are too sparse to investigate whether the kinetics are dose dependent. However, the half-life estimations in workers with initial higher (i.e., rubber workers; Figure 2) versus lower (i.e., dismantlers) BDE-209 levels seem to agree. A previous NONMEM calculation, based on data from the four dismantlers only (Table 2, subjects A–D), having initial BDE-209 levels much lower than the rubber workers, yielded the estimate of 7 days (95% CI, 3–16 days) (Sjödin 2000).

Experimental data are well in accordance with our observations. A rapid clearance of BDE-209 has been indicated in carp (Stapleton et al. 2004) and rats (El Dareer et al. 1987; Norris et al. 1975; Sandholm et al. 2003). The half-life calculated in gray seals, 8.5–13 days (Thomas et al. 2005), agrees well with our estimate of 15 days (95% CI, 11–18 days). From a theoretical point of view, it is also reasonable that BDE-209 should have a relatively shorter half-life than other PBDEs because of its susceptibility to undergo, for example, reductive dehalogenation and substitution reactions under experimental conditions (Eriksson et al. 2003; Rahm et al. 2005). Also, hexabromobenzene, another perbrominated aromatic compound, has been shown to be readily metabolized in the rat (Yamaguchi et al. 1988).

The general pattern of decreasing half-lives of PBDE congeners with increasing number of bromine substituents was thus expected. Within each group of isomers, such as the nona-BDEs and octa-BDEs, the estimated apparent half-lives varied nominally, albeit with wide and overlapping confidence intervals. It can be noted, however, that the PBDE congeners with the shortest half-lives within each group of isomers have a hydrogen substituent in one of the phenyl rings and the other ring fully brominated. No further conclusions can yet be drawn from these observations.

The metabolism of BDE-209 is not yet fully elucidated. In the rat, Mörck et al. (2003) reported that BDE-209 was metabolized, excreted, and only marginally distributed to adipose tissue, but it was found in plasma and blood-rich tissues. Moreover, traces of nona-BDEs were also observed. For octa- and nona-BDE, experimental data are entirely lacking. In rainbow trout, Kierkegaard et al. (1999) found a clear accumulation of nona- and octa-BDEs over time, after exposure to deca-BDE. On the other hand, studies of carp have shown a rather rapid degradation of BDE-209, but no accumulation of hepta-BDEs to nona-BDEs (Stapleton et al. 2004). It seems likely that nona-BDEs and octa-BDEs are formed in humans after exposure to BDE-209, as previously indicated in our cross-sectional study of rubber workers, using a technical deca-BDE product containing only trace levels of octa- and nona-BDEs (Figure 1) (Thuresson et al. 2005). Furthermore, our findings from rubber mixers investigated after a 1-day deca-BDE exposure (Figure 3) support metabolic formation of octa- and nona-BDEs from BDE-209.

There are yet no other half-life calculations based on observational human data. Estimates based on animal experimental data have been published previously for BDE-47, BDE-99, BDE-100, BDE-153, and BDE-154 (Geyer et al. 2004), indicating long half-lives for all these congeners. Their significant tissue burdens in both wildlife and humans further support the persistence of these PBDE congeners. Our observations for BDE-153 agree well with the estimated long half-life (Geyer et al. 2004), but we refrained from calculation because our observation period was too short.

BDE-209 has been shown to have developmental neurotoxicity in mice (Viberg et al. 2003), and its potential carcinogenicity as observed in the National Toxicology Program (NTP 1986) must also be considered. It is obvious that BDE-209 differs from low- to medium-brominated PBDEs because, despite its short half-life in blood, it is present in male Swedes representing the general public at levels similar to those of BDE-47 (Jakobsson et al. 2005; Thuresson et al. 2005). Taking the rapid turnover of this compound into account, humans must be exposed more or less continuously to keep up the blood concentration. It seems likely that BDE-209 is metabolized to nona-BDEs and octa-BDEs, but the metabolism is probably more complex, as indicated in an experimental study in the rat (Mörck et al. 2003), finding also hydroxylated metabolites that may be more toxic than the parent compound. BDE-209 and the other higher BDEs are of special concern because large amounts of deca-BDE are still used, while the penta- and octa-BDE products are phased out. Thus, better knowledge of the metabolism and kinetics of higher BDEs, along with a better understanding of their toxicology, is needed.

## Figures and Tables

**Table 1:** Median serum concentrations (range) of some PBDE congeners (pmol/g lipid weight) in occupationally exposed workers and referent groups (cross-sectional data).

Study group	M	F	BDE-47	BDE-153	BDE-183	BDE-203	BDE-209
Electronics dismantlers^a^	15	4	5.9 (< 1–47)	7.0 (3.2–19)	11 (3.1–26)	—	5.0 (< 0.3–9.9)
Rubber workers^b^	7	—	1.2 (< 1–3.7)	1.3 (< 0.5–3.2)	< 0.1 (< 0.1–1.1)	0.36 (< 0.2–1.3)	28 (1.2–140)
Computer clerks^a^	—	20	3.0 (< 1–10)	1.3 (0.80–5.1)	0.24 (< 0.02–1.4)	—	< 0.7 (< 0.3–8.0)
Hospital cleaners^a^	—	20	3.2 (< 1–34)	0.89 (0.64–7.6)	0.16 (0.025–0.39)	—	< 0.7 (< 0.3–3.9)
Abattoir workers^b^	17	—	2.5 (< 1–13)	2.9 (1.7–5.7)	< 0.1 (All subjects < 0.1)	< 0.2 (< 0.2–0.49)	2.5 (0.92–9.7)

Abbreviations: F, female; M, male.

a Data from Sjödin et al. (1999).

b Data from Thuresson et al. (2005).

**Table 2:** Serum concentrations (pmol/g lipid weight) of 10 PBDE congeners in eight subjects working either as electronics dismantlers or rubber mixers at different time points during their vacation (a time free from any work-related exposure).

				Serum concentration (pmol/g LW) of PBDE congeners									
Subject	Day of vacation	LW (g)	LC (%)	BDE-153	BDE-183	octa-1	octa-2	octa-3	BDE-203	BDE-206	BDE-207	BDE-208	BDE-209
Electronics dismantlers													
A	0	0.029	0.53	9.3	10	0.34	8.9	0.62	1.1	0.32	1.7	0.20	5.7
	4	0.032	0.55	10	10	0.37	8.9	0.55	0.97	0.19	1.3	0.17	2.8
	10	0.028	0.52	7.6	7.5	0.25	7.1	0.40	0.78	0.12	1.0	0.13	1.8
	17	0.027	0.53	9.5	8.3	0.27	7.3	0.36	0.71	0.12	0.94	0.13	1.5
	24	0.027	0.54	9.3	8.7	0.27	7.3	0.36	0.76	0.11	0.93	0.14	1.8
	28	0.027	0.52	8.3	7.5	0.20	6.4	0.30	0.58	0.091	0.84	0.12	1.5
	35	0.028	0.54	8.1	7.8	0.22	6.4	0.38	0.48	0.10	0.76	0.092	1.1
B	0	0.023	ND	7.0	8.7	0.31	6.6	0.79	1.1	0.26	1.5	0.21	2.9
	3	0.021	0.37	5.1	6.2	0.25	5.1	0.49	0.78	0.20	1.2	0.17	2.0
	10	0.023	0.40	6.1	7.1	0.28	5.6	0.57	0.72	0.15	1.1	0.16	1.6
	14	0.027	0.51	5.1	6.1	0.26	4.9	0.46	0.60	0.13	1.0	0.15	1.4
	21	0.032	0.57	5.0	5.2	0.20	4.2	0.37	0.55	< 0.06^a^	0.83	< 0.04^a^	1.6
	28	0.032	0.56	5.7	6.2	0.24	4.7	0.40	0.53	0.10	0.85	0.13	1.1
C	3	0.027	0.54	4.8	5.8	0.15	3.9	0.41	0.38	0.12	0.58	0.12	1.1
	6	0.026	0.55	4.7	5.5	0.17	3.9	0.42	0.38	0.11	0.55	0.12	1.2
	17	0.033	0.54	4.6	5.8	0.17	3.7	0.39	0.31	0.070	0.43	0.077	0.63
	26	0.030	0.50	4.4	5.1	0.14	3.3	0.35	0.26	0.067	0.35	0.056	0.90
	31	0.036	0.55	5.1	6.2	0.19	3.7	0.39	0.32	0.079	0.41	0.10	0.78
D	0	0.033	0.56	17	15	0.60	16	1.3	2.1	0.47	3.0	0.37	6.2
	4	0.032	0.54	15	11	0.38	9.6	0.68	1.1	0.22	1.6	0.20	3.5
	10	0.041	0.57	18	13	0.51	12	0.79	1.3	0.19	1.7	0.20	2.1
	17	0.042	0.57	18	12	0.35	8.6	0.86	1.1	0.17	1.1	0.14	2.7
	25	0.034	0.55	17	12	0.49	12	0.71	1.2	0.19	1.6	0.20	2.0
Rubber mixers													
E	0	0.054	0.98	3.2	1.1	2.7	14	3.2	1.3	6.9	11	2.3	140
	10	0.045	0.80	2.9	< 0.07^a^	2.5	17	3.5	1.2	4.2	9.2	1.9	95
	13	0.046	0.86	3.9	< 0.07^a^	2.8	15	3.2	1.1	2.8	9.3	1.5	110
	17	0.045	0.81	3.9	1.2	3.1	18	4.0	1.6	1.8	11	1.8	60
	24	0.059	1.0	7.6	1.7	2.1	26	5.0	1.7	3.1	10	1.5	50
	31	0.061	1.1	4.3	< 0.07^a^	1.7	11	2.5	0.80	1.2	4.2	0.58	19
	34	0.033	1.0	3.5	< 0.07^a^	2.9	17	3.8	1.4	2.4	11	1.5	48
F	0	0.045	0.78	1.7	< 0.07^a^	2.1	16	1.5	0.61	2.2	8.2	1.2	36
	10	0.040	0.76	1.0	< 0.07^a^	1.3	9.0	0.89	0.61	0.84	3.0	0.52	9.7
	13	0.056	1.1	1.8	< 0.07^a^	0.73	6.4	0.96	0.44	0.48	2.0	0.36	6.1
	17	0.046	0.81	1.6	< 0.07^a^	0.84	6.1	0.94	< 0.06^a^	0.46	1.8	0.33	6.6
	24	0.047	0.81	1.4	< 0.07^a^	0.72	5.6	0.80	< 0.06^a^	0.45	1.6	0.34	5.3
	31	0.043	0.78	1.4	< 0.07^a^	0.69	5.7	0.75	0.42	0.30	1.2	0.15	3.4
	34	0.040	1.0	1.1	< 0.07^a^	0.78	6.1	0.70	0.32	0.33	1.2	0.20	4.2
G	0	0.036	0.65	1.3	< 0.07^a^	0.69	5.0	0.57	0.47	0.99	3.7	0.69	28
	2	0.032	0.58	1.4	< 0.07^a^	0.83	6.0	0.73	0.64	1.5	4.8	1.6	67
	34	0.019	0.45	< 0.6^a^	< 0.07^a^	0.65	5.7	0.46	0.55	0.80	2.4	0.49	7.9
H	0	0.038	0.68	< 0.6^a^	< 0.07^a^	0.57	2.9	0.90	0.36	4.8	5.2	1.2	90
	9	0.036	0.65	0.99	< 0.07^a^	0.84	4.3	1.1	< 0.06^a^	2.1	4.4	0.64	33
	34	0.016	0.76	< 0.6^a^	< 0.07^a^	< 0.04^a^	2.4	< 0.04^a^	< 0.06^a^	0.72	1.7	0.33	6.6
LOQ				0.6	0.07	0.04	0.09	0.04	0.06	0.06	0.07	0.04	0.3

Abbreviations: LC, lipid content; LW, lipid weight; ND, not determined.

a Below LOQ.

**Table 3:** Calculated apparent half-lives of some PBDE congeners, based on observations during vacation from occupationally exposed electronics dismantlers and rubber mixers.

	*t*_1/2 (days)_			Baseline (all)			Addition electronics dismantlers			Addition rubber mixers			Addition abattoir workers and rubber millers		Samples with concentrations < LOQ (%)	
BDE congener	Est	SE	95% CI	Est	SE	CV %	Est	SE	CV %	Est	SE	CV %	Est	SE	Ce	CUe
BDE-209^a^	15	1.7	11–18	0.86	0.18	150	1.6	0.86	87	70	22	73	2.8	0.60	0	0.20
BDE-208^b^	28	5.5	17–39	0.045	0.012	ND	0.13	0.035	35	1.5	0.33	48	NA	NA	0.02	0.79
BDE-207^b^	39	17	4–73	0.36	0.067	50	0.63	0.54	96	7.5	2.5	54	NA	NA	0	0.12
BDE-206^b^	18	2.5	15–20	0.12	0.064	35	0.083	0.48	ND	3.3	2.6	56	NA	NA	0.02	0.42
Octa-1^b^	72	39	0–150	0.097	0.018	19	0.12	0.41	120	1.7	1.6	61	NA	NA	0.02	0.33
Octa-2^b^	85	28	29–140	0.35	0.14	130	3.6	2.7	130	12	4.2	54	NA	NA	0	0.63
BDE-203^b^	37	11	16–59	0.074	0.022	ND	0.055	0.15	1,700	0.21	0.37	366	NA	NA	0.09	0.87
Octa-3^b^	91	95	0–280	0.093	0.38	ND	0.44	0.52	43	2.4	1.7	69	NA	NA	0	0.21
BDE-183^a^	94	13	68–120	0.15	0.033	63	9.1	1.5	31	NA	NA	NA	NA	NA	0	0.61

Abbreviations: Ce, considered exposed; CUe, considered unexposed; CV %, relative coefficient of variation; Est, estimate; NA, not applicable; ND, not determined. Baseline estimates were derived from rubber millers and reference populations of cleaners, clerks, and abattoir workers. The model assumed that each subject had a certain non-work-related exposure that was constant but not dependent on profession. Each of the occupationally exposed workers also had a work-related exposure considered to be at steady state before vacation but different between groups and subjects.

a One hundred seven observations in 68 individuals.

b Sixty-seven observations in 28 individuals.
